# Orbitrap-Based Mass and Charge Analysis of Single Molecules

**DOI:** 10.1021/acs.accounts.3c00079

**Published:** 2023-06-06

**Authors:** Evolène Deslignière, Amber Rolland, Eduard H.T.M. Ebberink, Victor Yin, Albert J.R. Heck

**Affiliations:** # Biomolecular Mass Spectrometry and Proteomics, Bijvoet Center for Biomolecular Research and Utrecht Institute for Pharmaceutical Sciences, University of Utrecht, Padualaan 8, 3584 CH Utrecht, The Netherlands; ‡ Netherlands Proteomics Center, Padualaan 8, 3584 CH Utrecht, The Netherlands

## Abstract

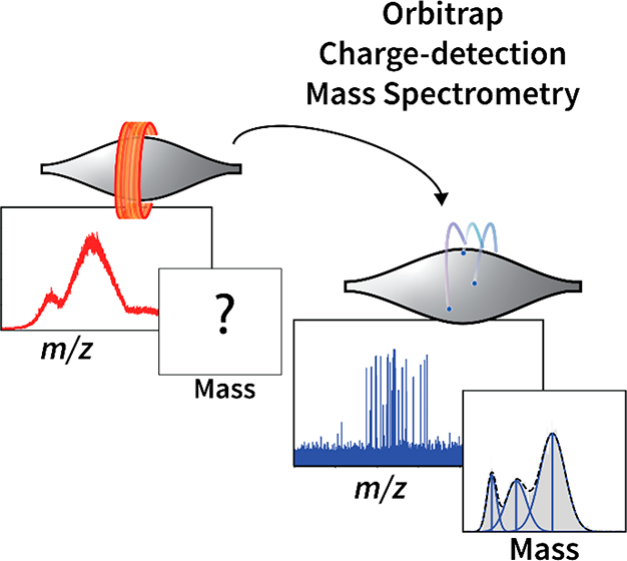

Native mass spectrometry is nowadays widely used for determining the mass of intact proteins and their noncovalent biomolecular assemblies. While this technology performs well in the mass determination of monodisperse protein assemblies, more real-life heterogeneous protein complexes can pose a significant challenge. Factors such as co-occurring stoichiometries, subcomplexes, and/or post-translational modifications, may especially hamper mass analysis by obfuscating the charge state inferencing that is fundamental to the technique. Moreover, these mass analyses typically require measurement of several million molecules to generate an analyzable mass spectrum, limiting its sensitivity. In 2012, we introduced an Orbitrap-based mass analyzer with extended mass range (EMR) and demonstrated that it could be used to obtain not only high-resolution mass spectra of large protein macromolecular assemblies, but we also showed that single ions generated from these assemblies provided sufficient image current to induce a measurable charge-related signal. Based on these observations, we and others further optimized the experimental conditions necessary for single ion measurements, which led in 2020 to the introduction of single-molecule Orbitrap-based charge detection mass spectrometry (Orbitrap-based CDMS). The introduction of these single molecule approaches has led to the fruition of various innovative lines of research. For example, tracking the behavior of individual macromolecular ions inside the Orbitrap mass analyzer provides unique, fundamental insights into mechanisms of ion dephasing and demonstrated the (astonishingly high) stability of high mass ions. Such fundamental information will help to further optimize the Orbitrap mass analyzer. As another example, the circumvention of traditional charge state inferencing enables Orbitrap-based CDMS to extract mass information from even extremely heterogeneous proteins and protein assemblies (e.g., glycoprotein assemblies, cargo-containing nanoparticles) via single molecule detection, reaching beyond the capabilities of earlier approaches. We so far demonstrated the power of Orbitrap-based CDMS applied to a variety of fascinating systems, assessing for instance the cargo load of recombinant AAV-based gene delivery vectors, the buildup of immune-complexes involved in complement activation, and quite accurate masses of highly glycosylated proteins, such as the SARS-CoV-2 spike trimer proteins. With such widespread applications, the next objective is to make Orbitrap-based CDMS more mainstream, whereby we still will seek to further advance the boundaries in sensitivity and mass resolving power.

## Key References

- WörnerT. P.; SnijderJ.; BennettA.; Agbandje-McKennaM.; MakarovA. A.; HeckA. J. R.Resolving Heterogeneous Macromolecular Assemblies by Orbitrap-Based Single-Particle Charge Detection Mass Spectrometry. Nat. Methods2020, 17, 395–398. 10.1038/s41592-020-0770-7.32152501(1)*In this paper, we introduce the principle of Orbitrap-based CDMS, highlighting its capacity to extract mass information from large, heterogeneous protein assemblies. We also describe the general workflow and suggest protocols for conducting these measurements.*
- WörnerT. P.; AizikovK.; SnijderJ.; FortK. L.; MakarovA. A.; HeckA. J. R.Frequency Chasing of Individual Megadalton Ions in an Orbitrap Analyzer Improves Precision of Analysis in Single-Molecule Mass Spectrometry. Nat. Chem.2022, 14, 515–522. 10.1038/s41557-022-00897-1.35273389PMC9068510(2)*In this fundamental study, we analyze the behavior of high-mass single particles inside the Orbitrap mass analyzer over the course of the detection period. We demonstrate how, using a segmented Fourier transform strategy, aberrations in the individual ion trajectories can be corrected and/or removed, greatly enhancing the quality of attainable mass information.*
- WörnerT. P.; SnijderJ.; FrieseO.; PowersT.; HeckA. J. R.Assessment of Genome Packaging in AAVs Using Orbitrap-Based Charge Detection Mass Spectrometry. Mol. Ther. Methods Clin. Dev.2022, 24, 40–47. 10.1016/j.omtm.2021.11.013.34977271PMC8671526(3)*In this work, we demonstrate how Orbitrap-based CDMS is particularly well-suited for the analysis of recombinant adeno-associated viruses (AAVs), an important gene delivery vector. Using Orbitrap-based CDMS, pharmaceutically critical attributes, such as the loaded genome mass and ratio of empty-to-filled AAV particles, can be accurately and efficiently obtained.*
- YinV.; LaiS.-H.; CanielsT. G.; BrouwerP. J. M.; BrinkkemperM.; AldonY.; LiuH.; YuanM.; WilsonI. A.; SandersR. W.; van GilsM. J.; HeckA. J. R.Probing Affinity, Avidity, Anticooperativity, and Competition in Antibody and Receptor Binding to the SARS-CoV-2 Spike by Single Particle Mass Analyses. ACS Cent. Sci.2021, 7, 1863–1873. 10.1021/acscentsci.1c00804.34845440PMC8577368(4)*Here, we use Orbitrap-based CDMS in conjunction with mass photometry to elucidate the detailed interactions between two extremely heterogeneous systems: intact IgG antibodies and a trimeric viral antigen (the Spike protein of the SARS-CoV-2 virus). The mass information we obtained provided unique insight into the complex, multivalent binding mechanisms underlying these antibody/antigen interactions.*

## Mass Spectrometry of Biomolecular Assemblies

Over the last decades mass spectrometry (MS) has been recognized as a central analytical technology for biomolecule characterization. Key to this development was the introduction of new ionization methods in the late 1980s, most notably matrix-assisted laser desorption ionization (MALDI) and electrospray ionization (ESI), jointly awarded the Nobel Prize in Chemistry in 2002.^5,6^ These ionization techniques provided an efficient method for charging molecules and bringing them into the gas phase, making them amenable to MS analysis.

Shortly after the introduction of ESI it was realized that, under appropriate conditions, even noncovalent protein complexes could be retained upon ionization, enabling MS to analyze biological, high-mass macromolecular assemblies.^7−10^ Such analyses, aptly named native MS,^11^ require samples to be electrosprayed under nondenaturing solutions, i.e., aqueous at physiological pH.

For high-speed and high-resolution analysis of ions generated from small molecules and protein digests (i.e., peptides) which occupy low *m*/*z* values (e.g., <2000), a variety of mass analyzers have been employed due to the relative ease of detecting analytes in this range.^12−16^ In contrast, folded intact proteins and their complexes, due to their larger mass (as well as adopting proportionally fewer charges per total mass), tend to exhibit higher *m*/*z* values.^17^ For high-resolution detection of these lowly charged protein assemblies, the Orbitrap has been the mass analyzer of choice, in part due to its favorable resolution scaling at high *m*/*z* values.^18^ This switch from small peptide analysis to large macromolecular ions comes with challenges in ion transmission and thus required specific technical modifications to be made to the Orbitrap mass analyzer.

**Figure 1:**
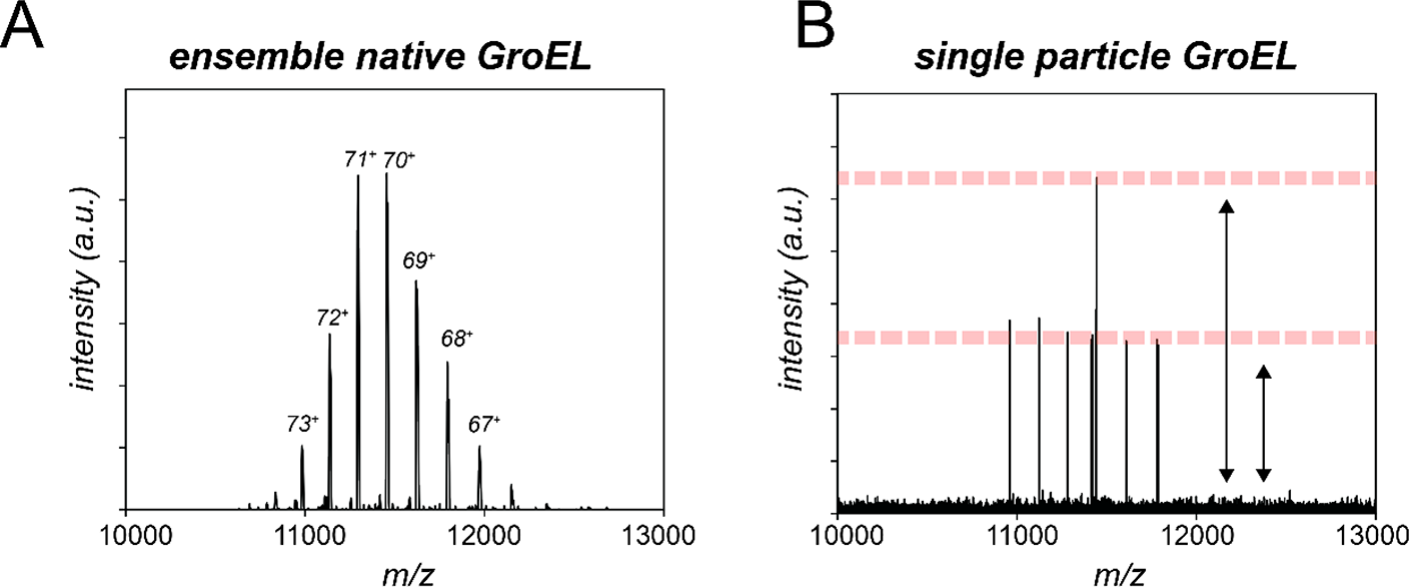
Transmission of macromolecular ions into the Orbitrap and different methods of mass measurement. (A) In standard, ensemble native MS experiments, millions of ions are simultaneously introduced into the Orbitrap mass analyzer, and the recorded image current is converted to an *m*/*z* spectrum. Signal accumulates for ions that bear the same mass and charge, resulting in a distribution of charge states. (B) In Orbitrap-based CDMS the number of ions entering the Orbitrap mass analyzer is reduced to 10^0^–10^1^ to avoid detecting multiple ions of identical mass and charge and the associated signal accumulation. Mass spectra recorded in the single-ion regime thus exhibit a “spike” for each ion detected, where the intensity relates to the ion’s induced image current (charge). Because charge is quantized, coincidental measurement of two ions of identical *m*/*z* in the same acquisition is distinguishable by the appearance of signals at ∼double intensity.

The first efforts to push the high-*m*/*z* detection limit on an Orbitrap based instrument (the Exactive Plus) was done by improved focusing of the macromolecular ions entering the MS while simultaneously promoting collisional cooling and desolvation once ions approached the Orbitrap.^19,20^ Trapping particles at the source to facilitate transfer from atmospheric pressure to ultrahigh vacuum and allow a delay in flight time of (slow) high-mass ions to the Orbitrap also had a positive impact on improving transmission efficiency.^21,22^ With these advances in place, high-mass ions could successfully be transmitted to the Orbitrap, resulting in high resolution spectra of intact protein assemblies, ranging from antibodies to higher mass complexes such as GroEL, proteasomes, and intact virus particles (Figure 1A). For high-resolution mass analysis by native MS, Orbitrap-based mass analyzers have been broadly embraced by the field as they enable relatively high mass accuracy and mass resolving power for any analyte with masses in between ∼50 Da and ∼18,000,000 Da.

A key concept in conventional MS is that molecular masses are not directly measured but are instead inferred from the mass-to-charge (*m*/*z*) ratios of the detected ions. For small ions at low *m*/*z* this task is relatively trivial due to their low number of charges and extremely high mass resolution, allowing isotopic resolution. However, intact protein ions typically exhibit a broad range of charge values, producing a so-called charge state distribution (CSD) (Figure 1A). For a single homogeneous protein, the mass can still be calculated in a straightforward manner from the *m*/*z*-spacing between consecutive peaks from the CSD.^23^ Correct charge state assignment becomes problematic, and is often the primary bottleneck to a successful experiment, as analytes exhibit higher masses and/or heterogeneity.^24^ First, the spacing between adjacent peaks in a CSD becomes smaller as analyte mass (and thus charge) becomes larger, decreasing the certainty of charge assignment. Second, compositional variations of related analytes arising from, e.g., isoforms, truncations, or post-translational modifications may greatly increase the number of peaks in each mass spectral region. As Orbitrap signals in a native MS experiment are commonly averaged prior to analysis, the resulting peaks in the mass spectrum will broaden, leading to the loss of mass resolving power and obfuscating individual charge peaks.

With applications of native MS moving away from the historical norms of purified recombinant proteins and toward progressively more complicated mixtures (e.g., plasma glycoproteins,^25^ membrane-embedded proteins,^26^ proteins with multiple ligands,^27^ vaccines and gene-delivery vectors^28^), there is a growing need to tackle the charge inferencing problem. One way to avoid problematic signal convolution may come from measuring each ion individually. If a technique had the requisite sensitivity to detect individual ions, while simultaneously providing independent determination of its charge, the mass of every ion could be obtained, regardless of their bulk heterogeneity. True “mass measurement” of complex, heterogeneous biological samples would then be possible.

### Single-Particle Mass Determination by Orbitrap-Based Charge Detection Mass Spectrometry

Single particle Orbitrap-based charge detection mass spectrometry (Orbitrap-based CDMS) detects single, individual ions within the Orbitrap analyzer, and utilizes a method to experimentally determine their charge, allowing the ion mass to be extracted without requiring any indirect charge inferencing at the ensemble level. Orbitrap-based CDMS translates the technology historically developed for charge determination of single ions on various modified, home-built mass analyzers (e.g., time-of-flight,^29^ electrostatic linear ion traps^30,31^) to the broadly used commercially available Orbitrap analyzer. For readers interested in a more detailed recounting of the rich history behind the development of CDMS on non-Orbitrap mass analyzers, we point to several excellent reviews in refs (32−34).

**Figure 2:**
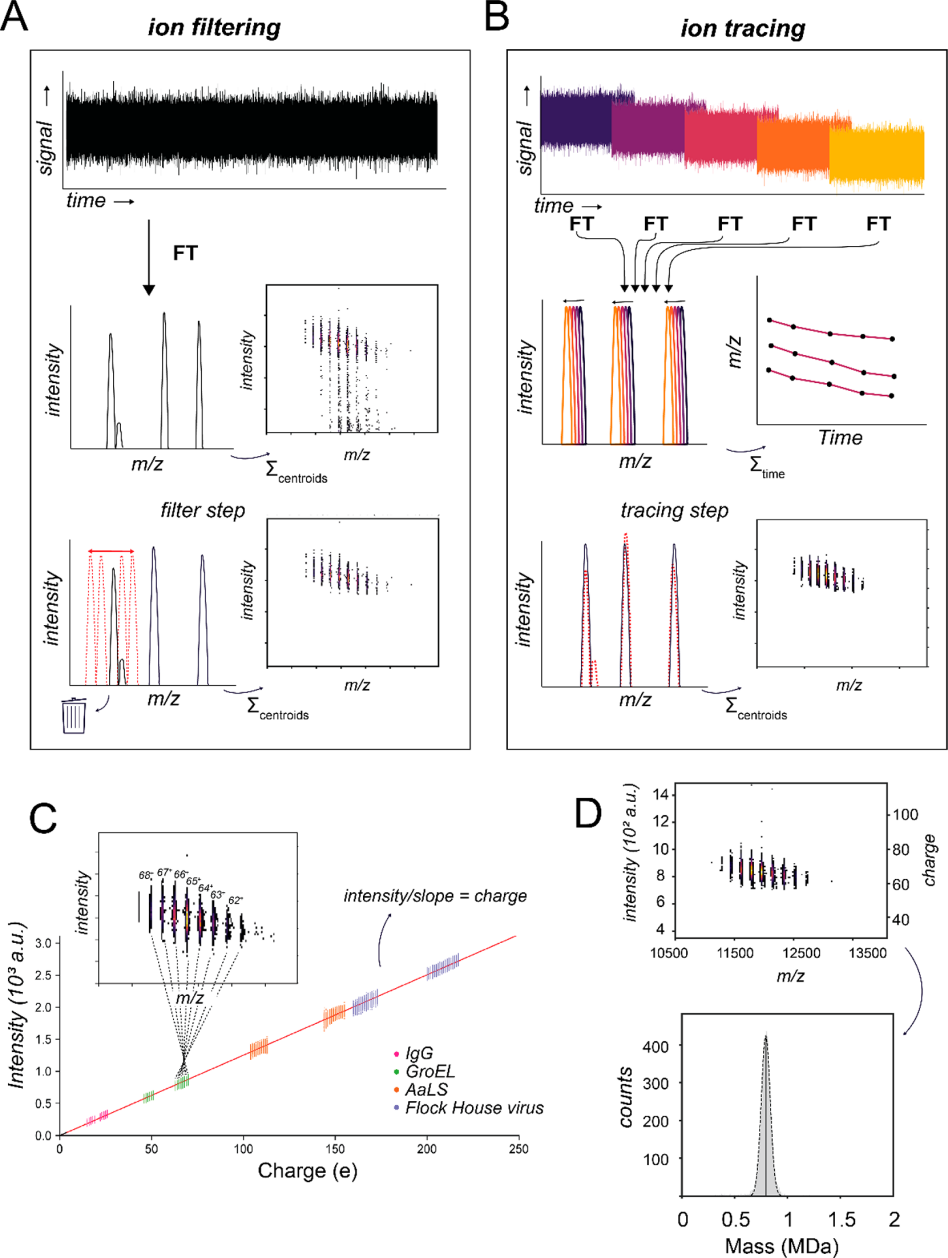
Processing of single-particle data in Orbitrap-based CDMS. (A) The single ion peaks are obtained in the frequency domain (=*m*/*z*) after FT of the image current collected in the time domain (the transient). For every peak both an *m*/*z* and intensity are recorded. During acquisition, some ions can drift in frequency, causing signal splitting and tailing to lower values in the intensity domain. Implementing a filter step in which split peaks are detected and eliminated, improving the determination of charge and thus also mass. This approach requires only the standard (frequency-domain) mass spectrum. (B) In the frequency-chasing approach, transients are segmented into time intervals, allowing ion drifts to be monitored over the detection period. Ions that would have previously been filtered can thus be addressed, enabling longer acquisitions and in turn improving the accuracy and precision of Orbitrap-based CDMS measurements. This approach requires access to the time-domain Orbitrap data. (C) The image current amplitude of an ion orbiting in the Orbitrap scales linearly with the ion charge. To determine this relationship quantitatively, single ion intensities of well-characterized biomolecules are plotted against their *a priori* determined charge values (i.e., by standard inferencing) to generate an intensity-to-charge calibration curve. Once calibrated, single ion intensities can be directly converted to a corresponding charge value. Adapted with permission from ref (1). Copyright 2020 Springer Nature. (D) Calibrated assignment of charge together with their *m*/*z* values allows the mass of each individual ions to be calculated. These individual ion masses can then be plotted together in a zero-charge mass histogram.

The invention of Orbitrap-based CDMS relied on two major breakthroughs. First was the development of modern Orbitrap mass analyzers with the sensitivity to detect single ions, as first demonstrated with myoglobin and the 14-mer complex GroEL.^35,36^ In these experiments, the intensities of the ion signals appeared quantized, with each step increase corresponding to an additional, integer number of individual ions being simultaneously detected at the same *m*/*z* (Figure 1B).^35^ Second was the observation that under these single ion detection conditions, the amplitude of the generated image current (reflected in the precise intensity value of each peak) for each individual ion signal scaled directly with its charge,^1^ thus providing an avenue of direct charge determination (Figure 2C).

In theory, adapting a standard native MS experiment (hereby referred to as “ensemble native MS”) to a single ion native MS experiment requires only reducing the amount of analyte signal such that in each scan, just a single ion enters the Orbitrap for a given *m*/*z* value. This can be achieved in several ways, e.g., diluting the analyte prior to ESI (providing a 1,000- to 1,000,000-fold gain in sensitivity), or by decreasing the ion injection time and/or purposely “detuning” the ion optics to sample less of the ion beam. In practice, however, a successful single ion native MS experiment requires a few additional considerations in comparison to ensemble ion measurements. One major difference is the optimal transient length (i.e., instrument resolution setting). In an ensemble native MS experiment, modest transient lengths (16–64 ms, corresponding to resolutions 3,125 to 12,500 at *m*/*z* 400) are generally employed to improve signal-to-noise and leave any microheterogeneity deliberately unresolved, facilitating charge state inferencing.^37^ In contrast, Orbitrap-based CDMS benefits from comparatively longer transient lengths (512–1024 ms, corresponding to the maximum resolution settings on current commercial Orbitraps) for several reasons: the increase of charge resolution as a function of transient length, the increased ease of reaching the single ion regime due to the increased number of FT bins, favorable scaling of *m*/*z* resolution due to the absence of isotopic beat patterns,^36^ as well as the overall increases in signal-to-noise assuming the ions survive the entire transient duration.^1^

One of the primary challenges in a single particle Orbitrap-based CDMS experiment is maintaining stable ion orbits within the Orbitrap mass analyzer to take full advantage of the transient length benefits described above. In ensemble native MS, an important source of signal decay during the detection period is dephasing of the ion cloud ensemble within the Orbitrap mass analyzer.^38,39^ In Orbitrap-based CDMS, this avenue of decay is largely eliminated due to the nonensemble nature of the experiment. Instead, the primary source of signal decay in Orbitrap-based CDMS is direct interactions of the individual ions with residual background gas molecules within the Orbitrap mass analyzer. As such, optimal gas pressures utilized in Orbitrap-based CDMS are comparatively lower than those used in ensemble native MS (∼10^–10^ vs 10^–9^ mbar, respectively), although the pressure optimum must be balanced to benefit also from collisional cooling, which we demonstrated is of particular importance for adequate transmission and desolvation of macromolecular ions.^3^

For large, macromolecular protein assemblies (*e.g.,* viruses and membrane protein complexes) collisions predominantly cause neutral solvent losses. Therefore, these ions can drift or “jump” in their orbiting frequency as their mass changes during the detection period. If these drifts become sufficiently large to be resolved (i.e., they shift into an adjacent FT bin), they will display multiple, split peaks in *m*/*z* with lower intensities than expected for their charge (Figure 1A).^1,2^ We determined that the extent of peak splitting is heavily influenced by (1) the amount and nature (e.g., N_2_ or Xe) of the collision gas leaking into the UHV region and (2) the traveled distance of ions in the Orbitrap (both of which increase the probability of collisions), as well as (3) the extent of particle desolvation prior to Orbitrap analysis (as incomplete desolvation provides more solvent adducts to be potentially lost).^2^ Fine-tuning such parameters can invoke a trade-off between the amount of peak-splitting and the final resolution of the single ion acquisition. Ideally, to improve accuracy, a long transient time is desired, while on the other hand ideal ion behavior is more easily achieved at shorter times.

Regardless of instrument optimization, some amount of ion destabilization will likely still occur. Our group has developed several methods to address this challenge. Initially, we developed an algorithm to identify unstable ion trajectories by detection of peak splitting events, which can be readily identified by scanning near the parent peak for any satellite peaks (Figure 2A).^1^ These ions can then be filtered out from the data set. An alternative approach, developed in parallel by Kafader and colleagues, is to selectively analyze the ion’s induced image current only up to a decay event.^40^ Up until this destabilization, the accumulated signal yields a slope unique for each mass and charge (STORI plot).^40^ Even if an ion does not survive the full acquisition time in the Orbitrap, the extraction of its STORI slope typically suffices to obtain its *m*/*z* and charge information.^41^ For smaller proteins and biomolecules, which are very efficiently desolvated prior to entry into the Orbitrap and thus lack the capacity to further shed solvent/neutral losses, this approach can be effective as premature ion fragmentation and subsequent signal loss renders prolonged single-ion oscillations unattainable.

More recently, we developed an algorithm that partitions the recorded Orbitrap image current into discrete time slices to monitor the orbiting frequency of individual ions (Figure 2B).^2^ In this approach, any changes in individual ion frequencies/intensities will be directly tracked, allowing peak splitting caused by shifts in *m*/*z* to be identified and corrected (Figure 2B). Our approach was inspired by the stepped fast Fourier Transform (FFT) strategies originally pioneered in the 1990s and early 2000s for Fourier transform ion cyclotron resonance MS.^42−44^ This “frequency chasing” algorithm greatly extends the feasible acquisition time of single ions within the Orbitrap, as the nonideal ion behavior more frequently observed at longer time scales can be rectified during signal processing. Using this approach, we tracked and analyzed single ions over Orbitrap acquisition times of several seconds, and consequently could more accurately extract *m*/*z* and charge due to the enhanced resolution and signal-to-noise. Enabled by this method, we found that large, megadalton ions can survive for exceptionally long durations (*e.g.,* several seconds, with distance traveled in the Orbitrap equal to 8.5 km) without signal decay.^2^ With the current frequency chasing tools at hand, peak splitting can be mitigated and multisecond measurement times can improve the accuracy of charge determination in Orbitrap-based CDMS to an uncertainty approaching a single charge.

### Applications of Orbitrap-Based CDMS for the Characterization of Biomolecular Assemblies

One of the most attractive features of Orbitrap-based CDMS is its capability to determine the mass of an ion without requiring a resolved charge state distribution. Therefore, for highly heterogeneous protein assemblies where charge state assignment of ensemble data is impaired by peak interference in the *m*/*z* dimension, single-ion measurements with Orbitrap-based CDMS offer an excellent alternative. Our group has leveraged this strength to study several macromolecular systems that have been historically outside the purview of ensemble native MS, offering novel biological insights that had previously been inaccessible.

#### Heterogeneous Glycoproteins: Antibodies and Immune Complexes

Antibodies or immunoglobulins (Ig) are highly heterogeneous due to their large size/mass (∼150 kDa for an IgG monomer, ∼1,000 kDa for an IgM pentamer/hexamer) and varying glycosylation profiles.^45,46^ Studying the interactions between antibodies and antigens, receptors, and other biomolecules is vital for our understanding of immunology but provides further challenges because binding partners can also be heterogeneously glycosylated or can interact with multiple different stoichiometries.

**Figure 3:**
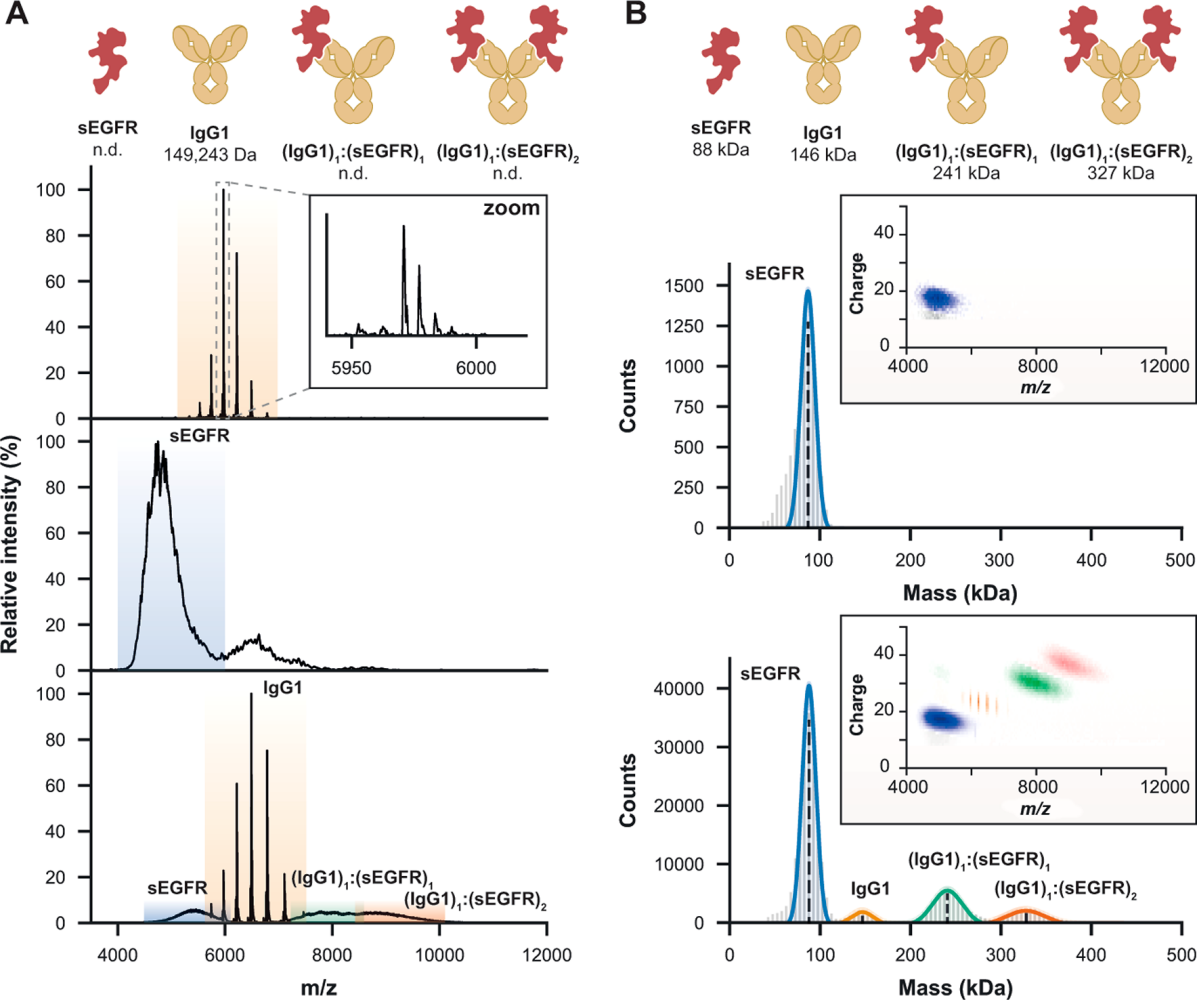
Mass determination of antibody–antigen complexes comparing ensemble native MS and Orbitrap-based CDMS. (A) Although ensemble native MS can resolve individual glycoforms of IgG1 (upper panel), the extensive glycosylation of the antigen sEGFR prohibits resolving features of sEGFR alone (middle) and of IgG1-sEGFR complexes (lower). (B) Orbitrap-based CDMS enables accurate mass determination of both sEGFR (upper) and of all co-occurring species of IgG1 bound to sEGFR (lower). Insets depict the two-dimensional separation in CDMS. Masses determined for each species, shown across the top of the panel, correspond to the mean (dotted line) of each fitted normal distribution. Adapted with permission from ref (48). Copyright 2022 American Chemical Society.

Ensemble native MS analysis of monomeric IgG-based antibody constructs is generally tractable due to their relatively simple glycosylation pattern,^47^ but higher extents of glycosylation on the antibody and/or antigen will eventually introduce sufficient heterogeneity to render this approach impractical. We recently compared multiple analytical techniques to determine the mass of several antibodies, their respective (glycosylated) antigens, and their corresponding immune complexes.^48^ One such antigen is the soluble domain of epidermal growth factor receptor (sEGFR), which has a protein backbone mass of just 69.4 kDa but contains 11 canonical N-glycosylation motifs that can be variably glycosylated.^49^ For sEGFR, no resolvable charge states were observed by ensemble native MS, rendering mass extraction impossible (Figure 3A). With Orbitrap-based CDMS, we determined not only the mass of glycosylated sEGFR (88 kDa, corresponding to ∼20 kDa of additional glycan mass) but also resolved complexes corresponding to two different stoichiometries (1:1 and 1:2) when sEGFR was incubated together with an anti-EGFR IgG1 monoclonal antibody (Figure 3B).

To further push the envelope on glycan heterogeneity, we also studied a mutant of the anti-EGFR IgG1, IgG1-RGY, which forms hexameric [IgG1]_6_ structures in solution, mimicking its active structure on a cell surface for complement activation.^50^ Due to this oligomeric arrangement, IgG1-RGY is capable of complexing with C1q, a multivalent subcomponent of the classical complement pathway. The mass of the IgG1 hexamer/C1q complex could be obtained using both ensemble native MS and Orbitrap-based CDMS due to its simple glycosylation status. Upon sEGFR antigen loading, only Orbitrap-based CDMS enabled mass determination of the fully formed ternary [sEGFR]_12_:[IgG1]_6_:[C1q]_1_ complex (2.42 MDa, containing over 100 putative glycosylation sites).

Finally, increasing even further in glycosylation, we demonstrated the benefits of Orbitrap-based CDMS by studying interactions of the trimeric SARS-CoV-2 Spike (S) protein with a library of antibodies targeting the spike protein.^4^ The backbone-only mass of the S protein trimer is 390 kDa, but with 66 glycosylation sites in total, the mass of the fully glycosylated trimer measured by Orbitrap-based CDMS is 477 kDa. Interactions between the S protein and antibodies are predicted to be extremely heterogeneous due to the multivalent interplay between an IgG1 (each containing 2 Fab arms capable of antigen interaction), and the trimeric S (which possesses 3 copies of each antigen). Orbitrap-based CDMS experiments revealed not only that IgG1 antibodies do not bind to the S trimer in the predicted ratio (3 antibodies per trimer) but also that the preferred binding stoichiometry of each clone differs. With Orbitrap-based CDMS we showed that these “partially-occupied” antibody-bound S trimers were incapable of engaging the host ACE2 receptor, even though not all antigenic sites were occupied.

When multiple oligomeric states coexist, overlapping isobaric charge peaks can already obfuscate information about the abundances of the different stoichiometries in ensemble native MS, which is worsened at even modest glycosylation levels, as exemplified by IgM antibodies. IgMs assemble into a complex mixture of oligomers in the absence of a joining chain (J-chain), and their heavy chains each contain 5 putative glycosylation sites.^51^ Despite overlapping in the *m*/*z* domain, signals for the oligomers can be orthogonally dispersed in the ion intensity (i.e., charge) domain using Orbitrap-based CDMS due to their different ESI charging behaviors.^1^ We could thus extract fully resolved CSDs for each oligomer. Masses could then be calculated using either the direct charge determination method of Orbitrap-based CDMS or a conventional charge inference method. In this case, the latter proved to be more accurate, with mass errors less than 1%. However, this approach was only enabled because we were able to first separate these three populations in charge using Orbitrap-based CDMS.

#### Adeno-Associated Viruses as Gene-Delivery Vectors for Gene Therapy

Adeno-associated viruses (AAVs) are small, nonpathogenic viruses capable of carrying a single-stranded DNA genome. AAVs have emerged as important gene delivery vehicles for gene therapy and vaccination. The tremendous potential of AAVs is well illustrated by their use in over 200 clinical trials, with half a dozen AAV-based gene therapy products currently approved by regulatory agencies.^52^ AAV capsids are assembled from three interrelated proteins: VP1, VP2, and VP3. Each capsid contains 60 copies of the three VPs with a variable VP1:VP2:VP3 stoichiometry, although it is generally assumed to be 5:5:50.^53^ A transgene can be bioengineered to be packaged into the AAV capsids to generate the gene-therapy product. A sizable fraction of produced AAV particles often lack the encapsulated vector genome, and are unable to act therapeutically. Other byproducts of DNA packaging include partially- (i.e., containing truncated DNA) and over-filled capsids (i.e., containing > one genome). Disparities in capsid composition and DNA packaging can result in heterogeneous AAV populations. The inherent complexity of AAVs combined with their high masses poses significant analytical challenges.^54^

**Figure 4:**
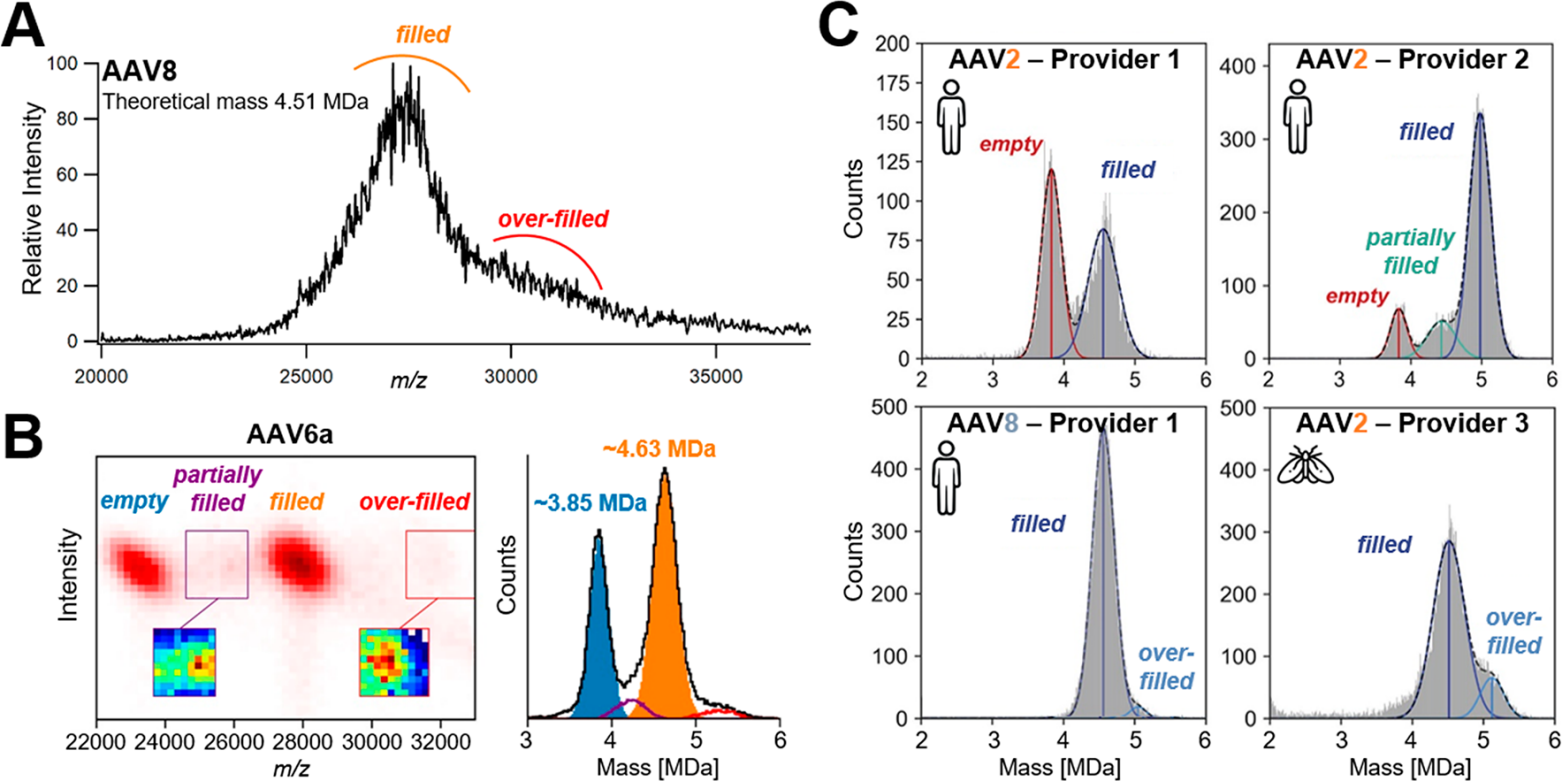
Characterization of the gene-delivery adeno-associated viral vectors (AAVs) using ensemble native MS and Orbitrap-based CDMS. (A) Ensemble native mass spectrum of AAV8 recorded on a UHMR Orbitrap (32 ms transient). Two populations, corresponding to filled and overfilled capsids, can be discerned, but precise mass determination is hampered by sample heterogeneity. (B) Two-dimensional CDMS histogram of an AAV6a sample (left) and its corresponding mass histogram (right). Adapted with permission from ref (3). Copyright 2022 Elsevier. (C) Mass histograms of genome-packed AAVs containing a CMV-GFP transgene measured by Orbitrap-based CDMS. Distinct mass distributions are observed across AAV2 samples produced either from human-cell or insect-cell based platforms and/or obtained from separate providers. Adapted with permission from ref (28). Copyright 2022 Elsevier.

In ensemble native MS experiments, direct charge state assignment is hampered by the large heterogeneity of AAV particles, particularly when genome-filled (Figure 4A). We demonstrated that Orbitrap-based CDMS could both resolve and measure the masses of empty (∼3.7 MDa) and filled (∼4.9 MDa, corresponding to the 3.8 kb transgene) AAV8 capsids.^1^ Experimental masses derived from Orbitrap-based CDMS were in excellent agreement with theoretical ones (mass deviation <1.2%), paving the way for the use of Orbitrap-based CDMS for AAV characterization.

Building on our initial proof-of-principle study, we subsequently developed an Orbitrap-based CDMS workflow aiming to monitor the integrity and amount of genome packed AAVs in a rapid manner, relying on direct sample dilution instead of buffer exchange.^3^ Our approach allowed for the detection and accurate quantification of not only empty and filled particles, but also of low abundance populations corresponding to partially filled (∼4.0–4.4 MDa) and overloaded (∼5.3 MDa) capsids for AAV6a samples (Figure 4B), as described previously by the Jarrold group for AAV8.^55,56^ By mixing predefined ratios of empty and filled capsids, we also pinpointed discrepancies between expected and experimental proportions. We further uncovered that seemingly pure filled samples can contain a substantial amount of partially filled particles, showing how Orbitrap-based CDMS is well-suited for rapid AAV quality control.

To further demonstrate the variability of AAV preparations, we next compared different AAV samples with a CMV-GFP transgene, produced either by using insect cell- or mammalian cell-based platforms, and provided by three distinct AAV producers (Figure 4C).^28^ When measuring genome-packed AAV2s, substantial differences were observed across all samples. For human-based AAV2s, the preparation of the first provider contained considerable amounts of empty particles. Conversely, the sample obtained from the second provider presented two low abundance subpopulations, i.e., empty and partially filled capsids (Figure 4C), the latter being particularly well resolved compared to what we previously measured (Figure 4B). Insect-based AAV2s from the third provider was the sole sample comprising overloaded species. The peak corresponding to filled capsids displayed a broader full width at half maximum than its human counterparts, suggesting an increased heterogeneity of insect-cell derived AAV2s. Lastly, our data set also clearly highlighted differences between AAV serotypes, as exemplified here by AAV2s vs AAV8s both produced by the same (first) manufacturer (Figure 4C). Similarly, O’Connor et al. showed lot-to-lot variability in AAV9 preparations.^57^ These studies emphasize the need for robust quality control methods to ensure consistency of vector composition and, more importantly, potency.

Although Orbitrap-based CDMS has been mostly employed to probe transgene-related attributes, its use can go beyond the characterization of capsid integrity and empty/filled ratios, even allowing VP heterogeneity to be tackled. In 2021, we proposed that AAV capsid assembly is stochastic, and native mass spectra are actually representative of multiple overlapping combinations of VP stoichiometries,^58^ generating complex spectral interferences. Orbitrap-based CDMS revealed that the spectral appearance of an AAV preparation is closely related to its ESI charging behavior, with lower charged AAVs correlated with more apparent heterogeneity.^59^ This suggests that Orbitrap-based CDMS could be an efficient tool to predict native MS distributions of AAVs. Overall, Orbitrap-based CDMS is particularly well-adapted for a thorough characterization of AAVs, which fall in a size regime where only a few analytical techniques are available. As such, CDMS is being rapidly adopted by several academic and industrial laboratories,^28,56,60−62^ with the technique even considered to be one of the gold standard methods to quantify capsid content.^63^

## Perspective and Future Outlook

In this Account, we highlight our developments and describe several recent applications of Orbitrap-based CDMS for biomacromolecular analysis. When compared directly to ensemble native MS, Orbitrap-based CDMS confers several distinct advantages, one of which is the ability to extract mass information from heterogeneous, non-charge state resolved systems. In this regard, we specifically highlight recent instances from our group targeting biological systems, where their inherent complexity and heterogeneity have until recently put them outside the reach of ensemble native MS, but within the reach of Orbitrap-based CDMS. The clearest advantage of Orbitrap-based CDMS is that its single molecule nature makes it inherently a million times more sensitive than ensemble MS. Several thousand individual ions are typically sufficient for a thorough statistical analysis from which mass distributions can be extracted. A particularly attractive feature of Orbitrap-based CDMS is its ability to be performed on any Orbitrap mass spectrometer without requiring any specialized hardware/software modifications or external equipment. This alone renders Orbitrap-based CDMS a fairly approachable technique with a low barrier of entry, as such mass spectrometers (*e.g.,* Orbitrap EMR/UHMR) are already widely employed for native MS analysis. Software tools such as UniDecCD have already been developed by the community to ease the analysis of Orbitrap-based CDMS data.^64^ The recent commercialization of a STORI-based software pipeline, marketed as Direct Mass Technology (DMT) mode, is also likely to facilitate integration of Orbitrap-based CDMS in academic and industrial environments.

We summarized our findings and considerations necessary to transition from ensemble native MS experiments successfully and practically into single-ion Orbitrap-based CDMS experiments. We hope that our observations and suggestions will inspire more widespread adaptation of Orbitrap-based CDMS among native MS practitioners and beyond.

An obvious (but perhaps rather loaded) question is: can Orbitrap-based CDMS completely replace ensemble native MS as a method of mass determination of proteins and their complexes? While this could certainly be the case in the future, in its current state of early maturation the answer is still dependent on the analytes of interest. For cases where charge state resolution is not possible, Orbitrap-based CDMS provides mass information that would otherwise be unobtainable via ensemble native MS. If charge state inferencing is still feasible (i.e., well-resolved peaks), then both ensemble native MS and Orbitrap-based CDMS can redundantly yield mass information. For now, the variation in measured ion intensity in Orbitrap-based CDMS under standard instrumental conditions is still larger than a single elementary charge under standard operating conditions (σ = 3 charges at 1024 ms transients),^1^ thereby introducing a degree of uncertainty in charge assignment, and thus also in mass. For these simple, idealized cases, ensemble native MS is still expected to provide higher resolution mass information. On the other hand, if correct charge state assignment remains ambiguous, then a level of mass uncertainty for ensemble native MS will manifest that will not be present using Orbitrap-based CDMS. Hybrid approaches can also be applied to bridge these two regimes, *e.g.*, separating isobaric signals by Orbitrap-based CDMS followed by subsequent conventional charge state inferencing, as we recently demonstrated in our mass analysis of mixtures of IgM tetramers to hexamers.^1^ The greatest benefit of Orbitrap-based CDMS may be its lower analyte concentration requirements vs ensemble native MS, which can be particularly advantageous in cases where sample quantities are limited. Here the sky may be the limit, if we find the technologies to bring single molecules directly from a cellular environment *one-by-one* to the mass analyzer without any sample losses, we could begin to assess the proteome of the cell with single copy resolution, providing information on all proteoforms present. Although a dream for now, the back-end sensitivity to detect these single molecules is already there.

Evidently, there exist many exciting avenues to further enhance the technical capabilities of Orbitrap-based CDMS as a mass analysis technique. One approach is to improve the resolution in the detected charge, which is still a major contribution to mass error (as Orbitrap mass analyzers provide excellent resolution in the *m*/*z* domain). Since charge resolution improves as a function of transient length,^2^ improvements in detection periods could theoretically enhance the mass resolution of Orbitrap-based CDMS by several-fold. Alongside resolution improvements, the applicability of Orbitrap-based CDMS would also benefit from coupling with online separation methods. The primary challenge to combat is the changes in analyte concentration over a chromatographic elution. To this end, recent developments in acquisition software for real-time ion density modulation of single ions may be highly beneficial.^65^ We anticipate that the following years will yield numerous exciting developments and applications of Orbitrap-based CDMS in providing unique insight into a wide arena of biological questions.
